# *NPC1L1 *haplotype is associated with inter-individual variation in plasma low-density lipoprotein response to ezetimibe

**DOI:** 10.1186/1476-511X-4-16

**Published:** 2005-08-12

**Authors:** Robert A Hegele, Justin Guy, Matthew R Ban, Jian Wang

**Affiliations:** 1 Vascular Biology Group, Robarts Research Institute, London, Ontario, Canada; 2 Department of Medicine, Schulich School of Medicine and Dentistry, University of Western Ontario, London, Ontario, Canada

## Abstract

**Background:**

*NPC1L1 *encodes a putative intestinal sterol transporter which is the likely target for ezetimibe, a new type of lipid-lowering medication. We previously reported rare non-synonymous mutations in *NPC1L1 *in an individual who had no plasma lipoprotein response to ezetimibe. We next hypothesized that common variants in *NPC1L1 *would underlie less extreme inter-individual variations in the plasma LDL cholesterol response to ezetimibe.

**Results:**

In 101 dyslipidemic subjects, we found that *NPC1L1 *haplotype was significantly associated with inter-individual variation in the response of plasma LDL cholesterol to treatment with ezetimibe for 12 weeks. Specifically, about one subject in eight lacked the common *NPC1L1 *haplotype 1735C-25342A-27677T and these subjects had a significantly greater reduction in plasma LDL cholesterol with ezetimibe than subjects with at least one copy of this haplotype (-35.9+4.0 versus -23.6+1.6 percent reduction, P = 0.0054). This was paralleled by a similar non-significant trend of between-haplotype difference in reduction of total cholesterol.

**Conclusion:**

These preliminary pharmacogenetic results suggest that *NPC1L1 *variation is associated with inter-individual variation in response to ezetimibe treatment.

## Background

Ezetimibe, the first member of a new class of medications, primarily reduces plasma concentration of low-density lipoprotein (LDL) cholesterol by blocking sterol absorption in enterocytes [1]. Ezetimibe probably interferes with the normal function of the *NPC1L1 *gene product, which appears to govern sterol absorption in the small intestine [2-5]. The mean plasma LDL cholesterol reduction seen with ezetimibe is 20 to 25%, and this has been remarkably consistent across patient subgroups defined by age, gender, ethnic background and concomitant use of other lipid regulating agents, such as statin drugs [6-9]. But despite the concordance in mean reductions, there is a wide range of inter-individual variation in the LDL cholesterol response to ezetimibe. A possible genetic basis for this inter-individual variation was suggested by our previous observation of rare non-synonymous *NPC1L1 *mutations in a non-responder to ezetimibe [10]. During the course of those studies, we identified several single nucleotide polymorphisms (SNPs) in *NPC1L1 *[10]. These SNPs have enabled assessment of common genetic variation at *NPC1L1*, which we hypothesized would underlie less extreme inter-individual variations in the plasma LDL cholesterol response to ezetimibe.

## Results

### Clinical and demographic data

**Figure 1:**
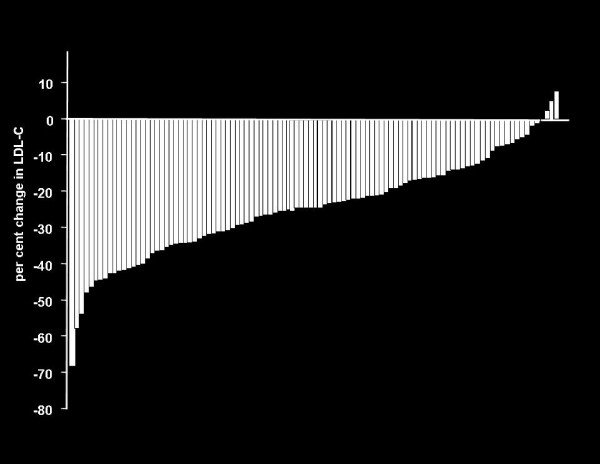
**Individual LDL-cholesterol response to ezetimibe 10 mg. **Each bar represents the percent change in LDL-cholesterol from baseline for one study subject; these data are arranged in rank order to show the range of responses.

Descriptive baseline clinical features are shown in Table 1. All subjects had baseline and follow-up fasting lipoprotein profiles measured. Mean follow-up was 84 days (12 weeks). None of the 101 subjects withdrew from treatment and compliance was excellent as judged by tablet counting. No adverse events were reported. Mean responses to ezetimibe treatment are shown in Table 2. The range of LDL cholesterol responses to ezetimibe treatment are shown in Figure 1. There was no difference in mean LDL cholesterol change in female versus male subjects or in subjects on ezetimibe monotherapy versus subjects on ezetimibe in combination with statin treatment.

**Table 1:** Baseline (mean ± standard deviation) and on-treatment clinical and biochemical attributes

	overall	males	females
number	101	61	40
age (years)	55.6 ± 11.9	54.0 ± 10.7	58.0 ± 13.3
days on treatment	83.8 ± 60.6	90.1 ± 67.6	74.5 ± 47.9
percent on statin	69.3	78.7	55.0
plasma lipids and lipoproteins (mmol/L)			
baseline			
cholesterol			
- total	6.57 ± 1.43	6.33 ± 1.54	6.93 ± 1.18
- LDL	4.51 ± 1.40	4.35 ± 1.53	4.72 ± 1.18
- HDL	1.18 ± 0.30	1.15 ± 0.29	1.22 ± 0.31
triglycerides	2.50 ± 2.36	2.63 ± 2.86	2.32 ± 1.33
			
on-treatment			
cholesterol			
- total	5.41 ± 1.32	5.10 ± 1.21	5.88 ± 1.35
- LDL	3.35 ± 1.15	3.21 ± 1.11	3.56 ± 1.19
- HDL	1.23 ± 0.45	1.16 ± 0.33	1.33 ± 0.60
triglycerides	2.25 ± 1.91	2.24 ± 2.12	2.25 ± 1.57

abbreviations: LDL, low-density lipoprotein; HDL, high-density lipoprotein;

**Table 2:** *NPC1L1 *genotype frequencies

SNP	genotype	number	frequency
1735C>G	C/C	56	0.55
	C/G	40	0.40
	G/G	5	0.05
			
25342A>C	A/A	54	0.53
	A/C	36	0.36
	C/C	11	0.11
			
27677T>C	T/T	66	0.65
	T/C	30	0.30
	C/C	5	0.05

abbreviation: SNP, single nucleotide polymorphism

### Genetic descriptors of study sample

*NPC1L1 *genotype frequencies are shown in Table 2. Allele frequencies were: 1) 0.75 and 0.25 for 1735C and 1735G, respectively; 2) 0.72 and 0.28 for 25342A and 25342C, respectively; and 3) 0.80 and 0.20 for 27677T and 27677C, respectively. Pairwise linkage disequilibrium correlation coefficients for SNP pairs 1735C>G:25342A>C, 1735C>G:27677T>C and 25342A>C:27677T>C were 0.24 (P = 0.017), 0.30 (P < 0.0001) and 0.44 (P < 0.0001), respectively. Thus, there was moderate but not strong linkage disequilibrium between these pairs of SNPs. Maximal likelihood haplotype definitions and frequencies are shown in Table 3. The most common haplotype (frequency ~0.6) was defined as 1735C-25342A-27677T and designated as "haplotype 2". For ANOVA, *NPC1L1 *haplotypes were collapsed to three groups, based on the presence or absence of haplotype 2. Thus, participants in this study had one of three possible diploid summary haplotypes: 2/2, 2/X or X/X, where X refers to any non-2 haplotype. There were 37, 51 and 13 subjects with haplotypes 2/2, 2/X and X/X, respectively.

**Table 3:** *NPC1L1 *haplotype definition and frequencies

designation	sequence definition	frequency
1	1735G-25342A-27677T	0.089
2	1735C-25342A-27677T	0.619
3	1735G-25342A-27677C	0.010
4	1735C-25342A-27677C	0.005
5	1735G-25342C-27677T	0.025
6	1735C-25342C-27677T	0.069
7	1735G-25342C-27677C	0.119
8	1735C-25342C-27677C	0.064

### Genetic associations with plasma lipoproteins

ANOVA (Table 4) showed no significant differences between baseline plasma lipoproteins. However, the percent change in LDL cholesterol on ezetimibe from baseline was significantly different between haplotypes (P = 0.02) and the percent change in total cholesterol from baseline on ezetimibe tended to be different between haplotypes. The significant between-haplotype differences from ANOVA were further explored assuming a dominant model for presence of haplotype 2, in which subjects with haplotypes 2/2 or 2/X were scored as "A" and subjects with haplotype X/X were scored as "B". In this model, subjects with haplotype X/X had a significantly greater percent reduction in plasma LDL cholesterol from baseline on ezetimibe than did subjects with haplotypes 2/2 or 2/X (-35.9 ± 4.0 versus -23.6 ± 1.6 percent, P = 0.0054). The range of reduction in LDL cholesterol in subjects with haplotype X/X was -68.2 to -19.6 percent. Furthermore, subjects with haplotype X/X tended to have a greater percent reduction in plasma total cholesterol from baseline on ezetimibe than did subjects with haplotypes 2/2 or 2/X (-22.8 ± 3.4 versus -15.9 ± 1.3 percent, P = 0.058).

**Table 4:** Clinical and biochemical features according to *NPC1L1 *haplotype

haplotype				
	2/2	2/X	X/X	P-value
number	37	51	13	
age (years)	56.6 ± 13.3	53.9 ± 11.3	58.4 ± 6.9	NS
percent female	48.6	37.3	38.5	NS
percent on statin	65.8	70.6	69.8	NS
baseline plasma lipids and lipoproteins (mmol/L)				
cholesterol				
- total	6.88 ± 1.33	6.31 ± 1.59	6.73 ± 1.01	NS
- LDL	4.64 ± 1.39	4.41 ± 1.51	4.49 ± 1.10	NS
- HDL	1.16 ± 0.34	1.19 ± 0.29	1.20 ± 0.21	NS
triglycerides	2.58 ± 1.75	1.99 ± 1.37	3.47 ± 3.78	NS
percent change on-treatment				
cholesterol				
- total	-16.5 ± 12.2	-15.8 ± 13.0	-22.8 ± 8.4	NS (0.07)
- LDL	-23.6 ± 13.2	-23.6 ± 14.7	-35.2 ± 13.5	0.02
- HDL	+10.7 ± 43.0	+1.2 ± 14.3	+0.6 ± 17.0	NS
triglycerides	-4.2 ± 35.6	+2.9 ± 44.9	-0 ± 30.3	NS

abbreviations: as in Table 1.

An additional *post hoc *analysis of individual SNPs found that the 11 homozygotes for the 25342C allele had a significantly greater percent reduction in plasma LDL cholesterol from baseline on ezetimibe than did other subjects (P = 0.02). Furthermore, the five homozygotes for the 27677C allele had a significantly greater percent reduction in plasma LDL cholesterol from baseline on ezetimibe than did other subjects (P = 0.013).

## Discussion

In this very preliminary analysis of a small sample of subjects with hypercholesterolemia, we found that genetic variation in *NPC1L1*, as defined by a three-site SNP haplotype, was significantly associated with inter-individual variation in the response of plasma LDL cholesterol to 12 weeks of treatment with ezetimibe 10 mg daily. Specifically, about one subject in eight did not carry the common *NPC1L1 *haplotype 1735C-25342A-27677T (designated "haplotype 2"); these subjects were found to have a significantly greater reduction in plasma LDL cholesterol with ezetimibe than subjects with at least one copy of haplotype 2 (-35.9 ± 4.0 versus -23.6 ± 1.6 percent reduction, P = 0.0054). This was paralleled by a similar non-significant trend of between-haplotype differences in reduction of total cholesterol. There were no significant between-haplotype differences in ezetimibe-related changes in plasma triglycerides or HDL cholesterol.

As with many association studies, the present study has limitations [11], including: 1) a small sample size; 2) no replication sample; 3) no demonstrated functional consequences of the *NPC1L1 *SNPs or haplotype; 4) no intermediate phenotype, such as cholesterol absorption; and 5) the potential that the positive findings were related to linkage disequilibrium with unmeasured markers at or near the *NPC1L1 *locus. Also, we did not genotype all SNPs at this locus. However, previous genomic screening experiments [10] indicated that the remaining SNPs were rare, and thus less likely to add information to the three SNPs studied. Inclusion of these rare SNP genotypes in the extended haplotype would have further subdivided the data into very small-sized cells for statistical analysis.

Our study confirms the similarity of the mean LDL cholesterol response to ezetimibe, namely a 20 to 25% reduction, in various study samples and across a range of demographic features including sex and concomitant statin treatment (6–9). Figure 1 demonstrates that this consistent mean LDL cholesterol reduction occurs on the background of relatively wide inter-individual variation in response. Our findings further indicate that subjects who carry the most common *NPC1L1 *haplotype (namely haplotype 2), have an ezetimibe-related LDL cholesterol reduction that is within the expected 20 to 25% range. However, a small but substantial group of subjects without haplotype 2 experienced a significantly greater LDL cholesterol reduction, on the order of 35%.

## Conclusion

The current finding of *NPC1L1*-associated inter-individual differences in LDL-cholesterol response to ezetimibe together with our earlier demonstration that rare missense mutations in *NPC1L1 *are associated with non-response to ezetimibe [10] support a relationship between this gene product and the mechanism of action of ezetimibe. Clearly, additional mechanistic and genetic studies are required. But these pharmacogenetic results, if confirmed, are consistent with the idea that the *NPC1L1 *is the ezetimibe target.

## Methods

### Study subjects

Between December 2003 and May 2004, 101 patients with primary hypercholesterolemia (defined as elevated LDL cholesterol) were treated with ezetimibe 10 mg daily according to national dyslipidemia guidelines [12]. Basic demographic attributes of study subjects are shown in Table 1. About one-third of patients were not taking any lipid-lowering medication and the remainder were stable on statin treatment of ≥12 weeks' duration prior to initiation of ezetimibe. Concomitant statin treatment included atorvastatin, rosuvastatin and simvastatin in 30, 28 and 12 subjects, respectively. All subjects provided informed consent and the study was approved by the Ethics Review panel of the University of Western Ontario (review number 07920E).

### Biochemical and genetic analyses

The lipoprotein profile after a 12 hour fasting period was determined before initiation of ezetimibe treatment and again after a mean follow-up of 12 weeks. Lipoprotein determinations were performed according to the Ontario Lipoprotein Proficiency Program standards and LDL cholesterol was calculated using the Friedewald-Levy-Fredrickson formula [13].

Genomic DNA was extracted and three common informative *NPC1L1 *SNPs from across the coding sequence were chosen for genotyping. Allele-specific genotyping methods were used [10]. For the genotype of exon 2 SNP 1735C>G (trivial name L272L, dbSNP number 2072183), we amplified a 381 bp fragment containing exon 2 using primers 5' GCT CAA CTT CCA GGG AGA CA and 5' AGC TTG TCA GAG AGG CTG G. This was followed by treatment with shrimp alkaline phosphatase (SAP) and *Exo*I to remove primers and unincorporated dNTPs, followed by ddNTP extension (SnaPShot, PE Applied Biosystems, Mississauga, ON) with primer 5' ATA GGC ATG AGC CAC TGC AC and analysis on a 3730 DNA Sequencer (PE Applied Biosystems, Mississauga, ON). For the genotype of intron 18 SNP 25342A>C, we amplified a 766 bp fragment containing intron 18 using primers 5' GCC CAG GTA GAA GGT GGA GTC and 5' CGT TGT TTG AGA CAT ACA TAG CTG. This was followed by treatment with SAP and *Exo*I to remove primers and unincorporated dNTPs, gel purification and ddNTP extension with primer 5' CTG CCT GAC ACC TGG CTC TGA and fragment analysis. For the genotype of exon 20 SNP 27677T>C (trivial name V1296V), we amplified the 558 bp fragment containing exon 20 using primers 5' GAA GCT TGG GCT GTG AAC A and 5' CCA CTA TGG GAG CAG AGG AG. This was followed by treatment with SAP and *Exo*I to remove primers and unincorporated dNTPs, gel purification and ddNTP extension (SnaPShot, PE Applied Biosystems, Mississauga, ON) with primer 5' TCT CTC CGC AGG GCC TGA CGT, and fragment analysis.

### Statistical analysis

Analyses were performed using SAS version 8.2 (Cary, NC), with a nominal level of significance defined as P < 0.05. Significance of the deviation of SNP genotype frequencies from Hardy-Weinberg equilibrium was assessed using chi-square analysis. Pairwise linkage disequilibrium between *NPC1L1 *alleles was determined using correlation coefficients as described [14]. Three-site maximal likelihood haplotypes were constructed using PHASE version 2.0 [15]. Analysis of variance (ANOVA) was used to identify significant sources of variation for quantitative plasma phenotypes, using F-values computed from type III sums of squares, which is most appropriate for unbalanced study designs. The dependent variables in ANOVA were percent change from baseline of plasma total, LDL and high-density lipoprotein (HDL) cholesterol and triglycerides. The independent variable in ANOVA was *NPC1L1 *haplotype, with age and sex as covariates within each model.

## Authors' contributions

Robert A. Hegele: experimental design, manuscript preparation

Justin Guy: data generation and interpretation, manuscript approval

Matthew R. Ban: data analysis, manuscript approval

Jian Wang: data analysis, editing, manuscript approval
